# Natural Products for the Treatment of Obesity, Metabolic Syndrome, and Type 2 Diabetes 2016

**DOI:** 10.1155/2016/9072345

**Published:** 2016-09-15

**Authors:** Menaka C. Thounaojam, Srinivas Nammi, Ravirajsinh Jadeja

**Affiliations:** ^1^Department Ophthalmology, Augusta University, Augusta, GA 30912, USA; ^2^School of Science and Health, Western Sydney University, Richmond, NSW 2751, Australia; ^3^National Institute for Complementary Medicine (NICM), Western Sydney University, Richmond, NSW 2751, Australia; ^4^Division of Gastroenterology/Hepatology, Department of Medicine, Medical College of Georgia, Augusta University, Augusta, GA 30912, USA

The prevalence of obesity, metabolic syndrome, and type 2 diabetes is continuously on rise due to modernization of life style and changing dietary habits. Use of herbal medicines for the treatment of metabolic diseases is a viable option and list of potential candidates is ever expanding. This 2016 edition of this special issue regarding natural products for the treatment of obesity, metabolic syndrome, and type 2 diabetes contains 8 articles accepted from a total of 21 submissions consisting of 6 research articles and 2 clinical studies.

B.-S. Ko et al. reported beneficial effect of a 70% ethanol Korean mistletoe (*Viscum album* coloratum) extract (KME-E) in *β*-cell function and hepatic insulin sensitivity. KME-E decreased epididymal fat mass by increasing fat oxidation and exhibited greater potentiation of first-phase insulin secretion than the partial pancreatectomized rats. KME-E also increased *β*-cell mass by increasing *β*-cell proliferation and decreasing its apoptosis. In the* in vitro* studies, betulin potentiated insulin-stimulated glucose uptake via increased PPAR-*γ* activity and insulin signaling in 3T3-L1 adipocytes, whereas oleanolic acid enhanced glucose-stimulated insulin secretion and cell proliferation in insulinoma cells. The authors concluded that ethanolic extract of KME has more beneficial potential than its aqueous extract.

In another study, antidiabetic and hypolipidemic potential of antcin K, a triterpenoid isolated from* Antrodia camphorata*, was evaluated. It was observed that AnK-treated mice had significantly lowered blood glucose, triglyceride, total cholesterol, and leptin levels. Further, antihyperglycemic and antihypertriglyceridemic effects of AnK were comparable to metformin and fenofibrate, respectively. AnK-induced phosphorylation of AMP-activated protein kinase (phospho-AMPK) expression in the muscle and liver resulted in significantly increased skeletal muscular membrane expression of glucose transporter 4 (GLUT4) and decreased hepatic glucose-6-phosphatase (G6Pase) mRNA levels. Furthermore, AnK treatment inhibited hepatic fatty acid synthase (FAS) and sterol response element binding protein-1c (SREBP-1c) levels and enhanced peroxisome proliferator-activated receptor *α* (PPAR*α*) expression.

H.-Y. Jung et al. evaluated efficacy of a polyherbal formulation (containing* Fomitopsis pinicola*,* Acanthopanax senticosus*,* Viscum album*, and* Allium tuberosum*), against high-fat diet- (HFD-) induced obesity. Treatment of HFD fed mice with this polyherbal formulation for 12 weeks reduced body and white adipose tissue (WAT) weights and occurrence of fatty liver. Additionally, the polyherbal formulation reduced serum lipids, leptin, and insulin levels along with hepatic lipids. It also suppressed lipogenic mRNA expression levels in WAT.

Y. Zhang et al. used metabolomic approach to evaluate efficacy of isoflavones rich extract of* Radix Puerariae* in HFD + streptozotocin-induced diabetes in rats. Eleven potential metabolite biomarkers related to coagulation, lipid metabolism, and amino acid metabolism were identified. In another study, the effect of* Miconia* sp. extract on mRNA expression of PPAR*γ* and activity of *α*-amylase and *α*-glucosidase were evaluated. The authors concluded that the ethanolic extract of* Miconia* sp. increased mRNA expression of PPAR*γ* and inhibited *α*-amylase and *α*-glucosidase.

A meta-analysis study was conducted by X. Wei et al. to evaluate therapeutic effect of berberine in the treatment of nonalcoholic fatty liver disease (NAFLD). Authors searched Embase, PubMed, Cochrane Library, and so forth until March 2016 for randomized controlled trials using berberine to treat NAFLD. Results from six randomized controlled trials comprising 501 patients showed significant efficacy of berberine in reducing lipids, blood glucose, and HbA1c in NAFLD patients. The authors concluded that berberine has positive efficacy on blood lipids, blood glucose, liver function, insulin resistance, and fatty liver condition of NAFLD patients.

A randomized, double-blinded, double-dummy, active-controlled, and multiple-dose clinical study compared the efficacy and safety of mulberry (*Ramulus Mori*) twig alkaloid tablet and acarbose in individuals with type 2 diabetes mellitus. 24-week treatment with alkaloid extract (SZ-A) and acarbose significantly decreased HbA1c and postprandial plasma glucose levels. However, the fasting plasma glucose levels were not significantly changed in both groups. Interestingly, 1 of 23 patients in SZ-A group (4.76%) and 5 of 15 patients in acarbose group (33.33%) suffered from gastrointestinal adverse events and hence authors concluded that SZ-A tablet is a more effective and safe therapeutic option for glycemic control compared to acarbose, in patients with type 2 diabetes.

J. Tian et al. conducted a clinical retrospective trial to access the efficacy and safety of a Chinese herbal decoction in treating outpatients with type 2 diabetes mellitus (T2DM). A total of 142 diabetes outpatients were enrolled in this clinical retrospective trial. All patients received the decoction for at least 6 consecutive months. Multiple linear regression analysis showed that the change of last visited HbA1c has a significant relationship with the baseline HbA1c, duration of diabetes, and body mass index (BMI). Both fasting and postprandial glucose levels were significantly decreased compared to the baseline. The Chinese herbal decoction also improved islet cell function with decreased HOMA-IR and increased HOMA-*β*. Triglycerides (TG) and blood pressure (BP) were decreased significantly at months 12 and 6, respectively. During the observation period, one subject developed diabetes kidney disease (DKD) and one developed diabetic peripheral neuropathy (DPN).

